# Bone Response to Titanium Implants Coated with Double- or Single-Stranded DNA

**DOI:** 10.1155/2018/9204391

**Published:** 2018-06-13

**Authors:** Nagahiro Miyamoto, Rina Yamachika, Toshitsugu Sakurai, Tohru Hayakawa, Noriyasu Hosoya

**Affiliations:** ^1^Department of Endodontology, Tsurumi University School of Dental Medicine, Yokohama, Japan; ^2^Department of Removable Prosthodontics, Tsurumi University School of Dental Medicine, Yokohama, Japan; ^3^Department of Dental Engineering, Tsurumi University School of Dental Medicine, Yokohama, Japan

## Abstract

We aimed to evaluate* in vivo* bone response and* in vitro *apatite formation to titanium (Ti) implants, coated with double-stranded DNA (DNA-d) or single-stranded DNA (DNA-s), and to compare the influence in different structure of DNA, double strand and single strand on bone response and apatite formation. The bone responses to multilayered DNA-d/protamine or DNA-s/protamine coating implants were evaluated after implantation into the extracted sockets of rat maxillary molars. Apatite formation on either coating surface after immersion in simulated body fluid (SBF) was evaluated using the quartz crystal microbalance (QCM) method. DNA-d/protamine and DNA-s/protamine coatings produced more roughened and hydrophilic surfaces than untreated Ti. Animal experiments showed that higher bone-to-implant ratios were achieved 3 and 6 weeks after implantation using DNA-d/protamine and DNA-s/protamine coatings compared with Ti. QCM measurements revealed that each coating contributed to significant earlier apatite formation in SBF. We conclude that both DNA-d/protamine and DNA-s/protamine coatings enhanced early bone formation. We suggest that a DNA-multilayer coating is useful for the surface modification of a Ti implant.

## 1. Introduction

Titanium (Ti) is widely used for dental implants. Diverse surface modifications to Ti dental implants, such as mechanical blasting, acid treatment, or application of hydroxyapatite coating, promote bone healing and enhance new bone formation [1–3]. Further, coating with biomacromolecules such as cell adhesion proteins enhances cell attachment, proliferation, and differentiation [4].

DNA serves as a biomaterial, irrespective of its nucleotide sequence. Cytokines such as bone morphogenetic protein (BMP) or antibiotics can intercalate and bind between base pairs in the grooves of stacked base pairs of a DNA strand, and DNA is less antigenic compared with other biomacromolecules [5–8]. DNA may therefore serve as a bone-guiding scaffold, because it contains numerous phosphate groups, which can bind calcium-containing compounds. For example, Fukushima et al. attempted to apply DNA as a biomaterial for bone reconstruction and developed several DNA complexes with polycations such as chitosan, polyamino acids, and protamine [9–14]. They found that the DNA/protamine complex enhances bone regeneration after implantation into calvarial defects of rats [15–17].

Another example of an attempt to use DNA as a coating material for Ti implants was published by van der Beucken et al. who used an electrostatic self-assembly layer-by-layer technique to coat DNA with poly(allylamine hydrochloride) (PAH) or poly-D-lysine (PDL) [18]. They found that a multilayered DNA/PAH or PDL coating after immersion in simulated body fluid (SBF) affects the differentiation of osteoblast-like cells by increasing the deposition of osteocalcin [19]. Multilayer coating of DNA using a layer-by-layer technique with bis-ureido-surfactants (BUS) is histocompatible and favors early bone responses after implantation into the femoral condyles of rats [20].

Sakurai et al. examined the biological effects of multilayered DNA/protamine-coated titanium implants [21]. They applied a 2,2,2-trifluoroethanesulfonyl chloride- (tresyl chloride-) activated method to immobilize protamine onto Ti. The tresyl chloride method chemically immobilizes proteins onto a Ti surface [22, 23]. Protamine is initially immobilized on the Ti implant using the tresyl chloride-activated method, and the DNA/protamine multilayer is then coated using a layer-by-layer technique. They implanted DNA/protamine-coated implants into the extracted sockets of the maxillary molars of rats and found that this coating promotes new bone formation at the early stage of bone healing [21]. In these studies, double-stranded DNA (DNA-d) was used as a coating material. In contrast, single-stranded DNA (DNA-s) is commercially available as a nutritional supplement.

In the present study, we inserted DNA-d/protamine- or DNA-s/protamine-coated implants into the extracted sockets of rat maxillary molars and compared the bone response to Ti implants coated with DNA-d or DNA-s. Moreover, we evaluated apatite formation on a surface coated with DNA-d/protamine or DNA-s/protamine after immersion in SBF. To evaluate apatite deposition in SBF, we employed a quartz crystal microbalance (QCM) method [24] that detects adsorbed or deposited substances at a nanoscale level by monitoring the differences in the oscillation frequency of the quartz [25]. We aimed to evaluate the influence of DNA-d or DNA-s coatings to Ti implant in* in vivo* bone response and* in vitro *apatite formation.

## 2. Materials and Methods

### 2.1. Materials

**Figure 1:**
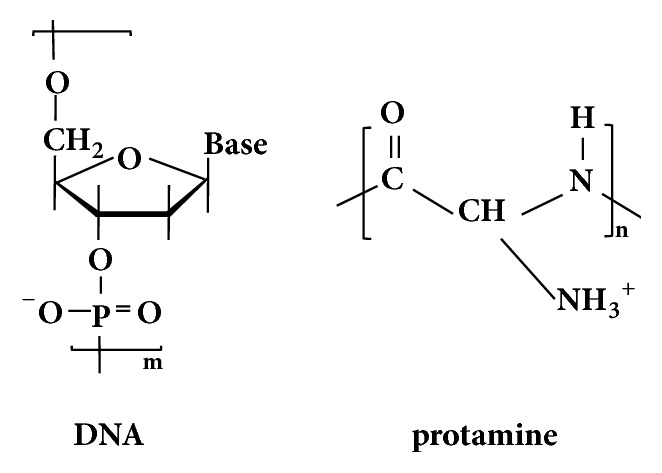
Structures of DNA and protamine.

DNase-cleaved DNA-d (300 bp; Maruha-Nichiro Corp., Tokyo, Japan), DNA-s (average molecular weight = approximately 55,580; Maruha-Nichiro Corp., Tokyo, Japan), and protamine (protamine sulfate, average molecular weight: approximately 4,500; Maruha-Nichiro Corp., Tokyo, Japan) were obtained from salmon testis (Figure 1). All materials were used without further purification. Ti disks (12.0 mm diameter, 1.0 mm in thickness; Japan Industrial Specification H4600, 99.9 mass% Ti; Furuuchi Chemical Corp., Tokyo, Japan) were used to characterize DNA-d/protamine and DNA-s/protamine multilayer coatings. Cast screw-type Ti implants (1.5 mm, diameter, 3.0 mm length; 99.8 mass% Ti; APEX Co., Tokyo, Japan) were used for animal experiments.

### 2.2. DNA-d/Protamine and DNA-s/Protamine Multilayer Coating

**Figure 2:**
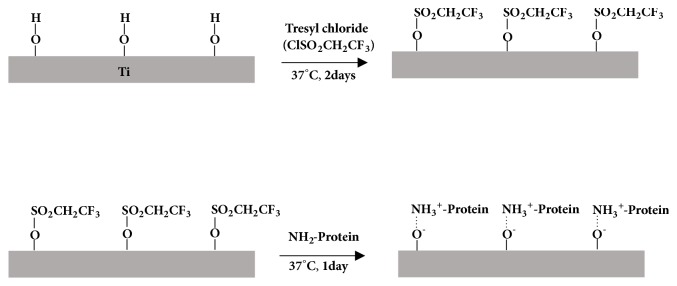
Schematic of protamine immobilization on Ti using tresyl chloride-activated method.

**Figure 3:**
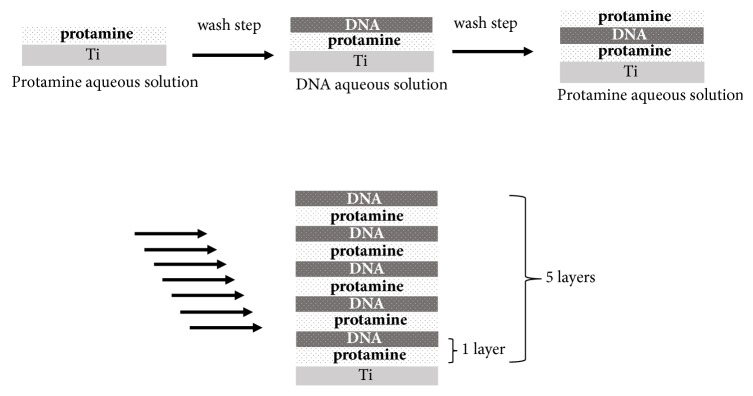
Schematic of the layer-by-layer coating technique for DNA and protamine.

Ti disks were cleaned with ethanol and then UV-irradiated before coating. First, protamine was immobilized on Ti disks using the tresyl chloride-activated method [22, 23] (Figure 2). Briefly, Ti disks were completely covered with tresyl chloride (Fluka, Buchs, Switzerland) and stored at 37°C for 2 days. After rinsing with double-distilled water, tresylated disks were immersed in an aqueous solution of protamine sulfate (1.7 mg/ml) for 24 h. The disks were then washed with double-distilled water to remove unreacted, overlaying protamine and excess tresyl chloride. The surfaces of the disks were alternately immersed in an anionic aqueous solution of DNA-d or DNA-s (1.0 mg/mL each) and a cationic polyelectrolyte protamine solution (1.7 mg/mL), for 7 min each, and then washed with double-distilled water at intervals according to previous reports [18, 21]. The accumulation of a multilayered DNA-d/protamine or DNA-s/protamine coating was continued until five protamine and DNA-d or DNA-s double layers were formed, with a top DNA layer (Figure 3) [21].

Using the same method, the surface of a screw-type Ti implant was coated with multilayered DNA-d/protamine or DNA-s/protamine after protamine immobilization. Each multilayer coated or uncoated screw-type Ti implant was sterilized using ethylene oxide gas before performing experiments with animals.

### 2.3. Fourier Transform-Infrared Spectroscopy (FT-IR) Measurement

The surfaces of DNA-d/protamine and DNA-s/protamine coatings were examined using FT-IR (FT-IR620, JASCO, resolution: 4 cm^−1^) with the Attenuated Total Reflection method using Ge prism.

### 2.4. Atomic Force Microscopy (AFM) Observation

The surface topographies of untreated Ti, DNA-d/protamine, and DNA-s/protamine disks were analyzed using an easy-Scan 2 FlexAFM (Nanosurf AG, Switzerland). Scans were performed in contact mode using a monolithic silicone probe coated with aluminum (Tap 190 A1-G, Budget Sensors; Innovative Solutions Bulgaria Ltd., Bulgaria). The typical scan was 2 *μ*m × 2 *μ*m. The surface roughness (Sa) of each sample was determined by AFM surface analysis.

### 2.5. Contact Angle Measurements

Contact angles of untreated Ti, DNA-d/protamine, and DNA-s/protamine surfaces versus double-distilled water were measured using a contact angle meter (DMe-201; Kyowa Interface Science Co. Ltd., Tokyo, Japan). The water-drop volume was maintained at 0.5 ml, and three 10 s measurements of each surface were made. Measurements were performed at 25 ± 1°C and 45 ± 1% humidity.

### 2.6. Implantation Procedure

The Animal Experimental Ethics Committee of Tsurumi University School of Dental Medicine approved this study (certificate no. 28A043). Animal experiments were performed according to the method reported by Raita et al. [26]. Twenty-four male Wistar rats (180 g, 6 weeks old) were housed, two per cage, at 20°C to 25°C under a 12 h circadian light rhythm, and fed a powdered diet and tap water* ad libitum* during the experiment. Each rat received one implant, and the 24 implanted rats were allocated to the groups as follows: untreated Ti (n = 4), DNA-d/protamine (n = 4), DNA-s/protamine for 3 weeks (n=4), untreated Ti (n=4), DNA-d/protamine (n = 4), and DNA-s/protamine (n = 4) for 6 weeks.

Surgery was conducted under general anesthesia induced using an intraperitoneal injection of ketamine hydrochloride (0.8 mg/kg) and medetomidine hydrochloride (0.4 mg/kg). The right-maxillary first molar was extracted using forceps. The socket of the mesial root of the right molar was enlarged using a dental reamer (#90-#150) after incision of the periodontal tissue. The screw-type implant was fixed into the enlarged root socket with a screwdriver. Incisions into periodontal tissue were closed with 7-0 polyamide nonabsorbable sutures (BioFit-D, WASHIESU, Tokyo, Japan). After surgery, rats were subcutaneously injected with benzyl penicillin G procaine (3,000,000 U/kg) and awakened with an intraperitoneal injection of atipamezole hydrochloride (0.2 mg/kg).

Rats were subcutaneously injected with the fluorochrome-labeling compound calcein (5 mg/kg; Wako Pure Chemical Industries, Ltd., Osaka, Japan) 1 week before sacrifice, namely, 2 or 5 weeks after surgery, to monitor new bone formation. Calcein is a stain that fluoresces after chelating calcium ion. Thus, activities of mineralizing bone surfaces can be monitored by calcein labeling. Animals were euthanized using a lethal dose of carbon dioxide gas 3 or 6 weeks after implantation. Each implant site, including the implant and bone tissue, was dissected using a diamond saw (Cutting Grinding System, BS-300CP band system; EXAKT, Apparate-bau GmbH & Co., KG, Norderstedt, Germany).

### 2.7. Histological and Histomorphometrical Observations

**Figure 4:**
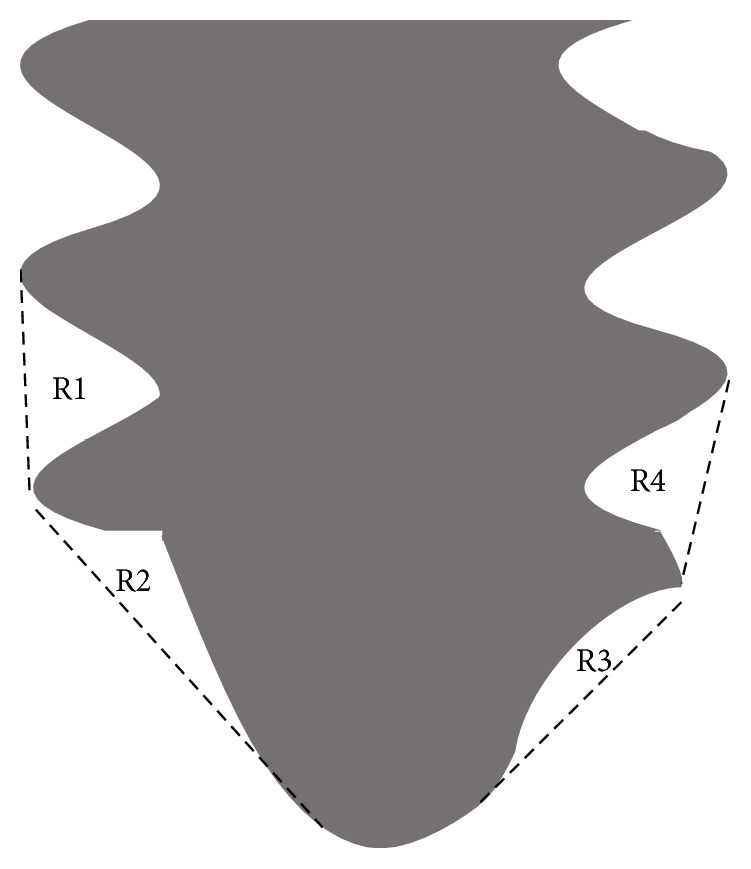
Illustration of the region of the interest (ROI) selected for histomorphometrical analysis.

Specimens were fixed in 10% neutral-buffered formalin for 7 days, dehydrated through a series of ethanol concentrations (70%, 80%, 90%, 96%, and 100%), and embedded in methylmethacrylate resin. After polymerization, nondecalcified sections were prepared using a cutting grinding technique (Cutting Grinding system, BS-300CP band system EXAKT; 400CS micro-grinding system; Apparatebau GmbH & Co., KG, Norderstedt, Germany) [27]. The thickness of the specimens was adjusted to approximately 50–70 *μ*m. Images were acquired using a confocal laser scanning microscope (CLSM) (TCS Multi-Photon, Leica, Germany) before staining the nondecalcified thin sections. Quantitation of new bone formation was identified using calcein labeling. The region of interest (ROI) for quantitative analysis of calcein labeling is the sum of R1, R2, R3, and R4 (Figure 4). The length of calcein labels per ROI in the CLSM images was measured using an image analysis system (WinROOF; Visual system Division, Mitani Corporation, Tokyo, Japan), and the total length of calcein was the sum of the lengths of each calcein label. After CLSM observations, nondecalcified thin sections were stained with methylene blue and basic fuchsin and observed using a light microscope (200×; BX51; OLYMPUS, Tokyo, Japan). Histomorphometrical analysis of the percentage of the bone-to-implant contact (BIC) values was conducted using an image analysis system. The BIC value was defined as the percentage of the implant length of the direct BIC value to the length of the screw implant in the ROI.

### 2.8. QCM Measurements

A 27-MHz QCM (AT cut, shear mode; AFFINIX QN*μ*; ULVAC Inc., Kanagawa, Japan) with a 500 *μ*L cell was used. The temperature was maintained at 25 ± 1°C, and the SBF solution in the cells was stirred at 1,000 rpm during measurements. The Ti sensor was prepared using sputtering deposition (CS200, ULVAC Inc., Kanagawa, Japan) under argon gas with a Ti target. The Ti sensor was cleaned using UV irradiation (BioForce Nanosciences Holding Inc., USA) for 20 min. Next, using the same method described above, the DNA-d/protamine or DNA-s/protamine multilayer coating was applied to the Ti sensor. Hanks' balanced salt solution (HBSS) without organic species was employed as the SBF [28]. The ion concentrations (mmol/L) of HBSS were as follows: Na^+^, 142; K^+^, 5.81; Mg^2+^, 0.811; Ca^2+^, 1.26; C1^−^, 145; HCO_3_^−^, 4.17, HPO_4_^2−^, 0.778; and SO_4_^2−^, 0.811.

**Figure 5:**
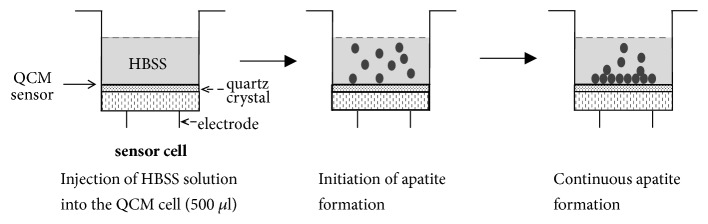
Procedures for QCM measurements for apatite formation in SBF.

The procedure for QCM measurement is illustrated in Figure 5. A sensor cell containing untreated Ti, DNA-d/protamine, or a DNA-s/protamine multilayer-coated sensor, was mounted on the QCM apparatus. SBF solution (500 mL) was injected into the cell. The frequency was monitored for 10 h after injection of the SBF solution. Apatite formation on each sensor decreases the frequency. The time when the frequency began to decrease was recorded, and the amount of apatite formation on each sensor 10 h after SBF injection was calculated using Sauerbrey's equation [29]. In a 27-MHz QCM system, a 1-Hz frequency decrease corresponds to a mass change of 0.61 ng/cm^2^ on the sensor.

### 2.9. Wavelength Dispersive X-Ray (WDS) Analysis of the Surfaces of Ti, DNA-d/Protamine, and DNA-s/Protamine Coatings after the Immersion in SBF

The sensor surfaces of Ti, DNA-d/protamine, and DNA-s/protamine coatings after the immersion in SBF were evaluated by wavelength dispersive X-ray analysis (WDS)(JXA-8900RL, JEOL Ltd., Tokyo, Japan) at an accelerating voltage of 20 kV by detecting the X-ray intensity of Ca-K*α*, P-K*α*. Elementary mappings of Ca and P were performed. The specimens were sputter-coated by gold before analysis.

### 2.10. Statistical Analysis

Data for surface roughness, contact angles, calcein labels, and BIC and QCM data for the Ti, DNA-d/protamine, and DNA-s/protamine coatings were analyzed using one-way analysis of variance and post hoc Tukey's test for multiple comparisons among means. An unpaired* t *test was used to compare date between calcein labels and the BIC after 3 and 6 weeks for each implant. Statistical analyses were conducted using Origin Pro 9.0 J (OriginLab Corp., Northampton, MA, USA). P <0.05 was considered significant, and data are expressed as the mean ± standard deviation (SD).

## 3. Results

### 3.1. FT-IR, AFM, Sa, and Contact Angles

**Figure 6:**
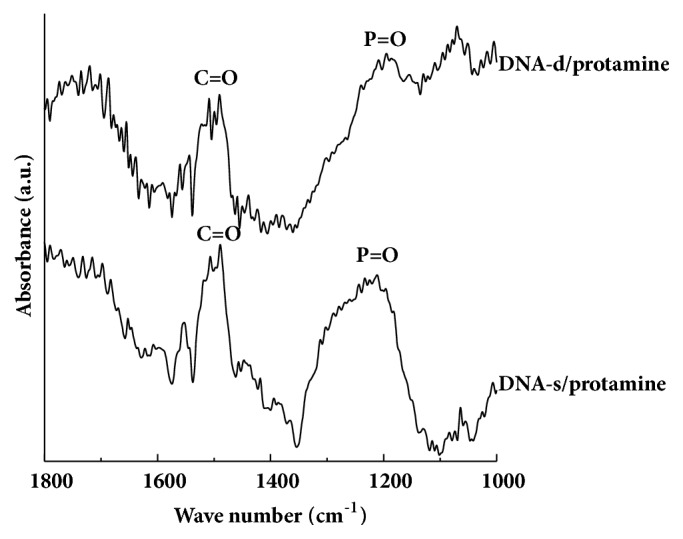
FT-IR spectrum of multilayered DNA-d/protamine and DNA-s/protamine coating surfaces.

FT-IR spectra of DNA-d/protamine and DNA-s/protamine coating surfaces are shown in Figure 6. Peaks around 1200 cm^−1^ were identified as the phosphate groups of DNA, and peaks around 1500 cm^−1^ were derived from the amide groups of immobilized protamine. Both DNA coatings clearly confirmed the presence of phosphate and amide groups.

**Figure 7:**
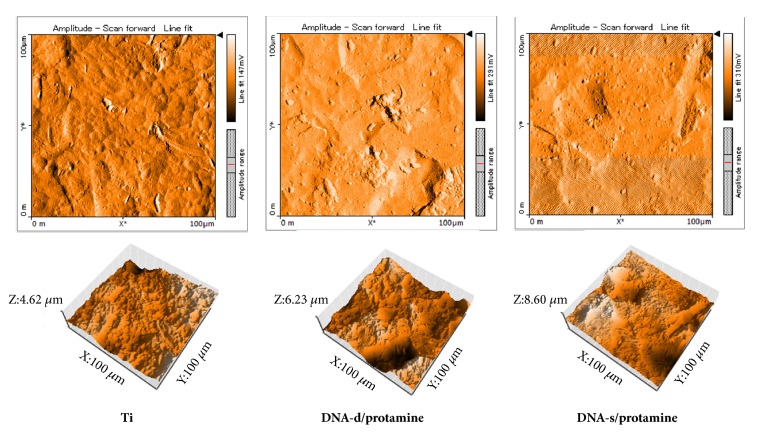
AFM images of Ti, DNA-d/protamine, and DNA-s/protamine surfaces.

AFM images of Ti, DNA-d/protamine, and DNA-s/protamine disk surfaces are shown in Figure 7. Surface appearances differed before and after DNA-d/protamine or DNA/s-protamine coating. Globular DNA-d/protamine or DNA/s-protamine coating was observed on DNA-coated disks, and no distinct differences were identified between DNA-d/protamine and DNA/s-protamine coatings.

Table 1 shows the Sa and contact angle for each sample by AFM surface analysis. AFM analysis enables it to measure roughness with nanoscale spatial resolution. The Sa values of DNA-d/protamine and DNA-s/protamine were significantly higher compared with that of Ti (*p* < 0.05), and the Sa values of DNA-d/protamine were significantly higher compared with that of DNA-s/protamine (*p* < 0.05). The contact angles of DNA-d/protamine and DNA-s/protamine were significantly smaller compared with that of Ti (*p* < 0.05). There were no significant differences between the contact angles of DNA-d/protamine and DNA-s/protamine (*p* > 0.05).

### 3.2. Fluorescent Labeling

**Figure 8:**
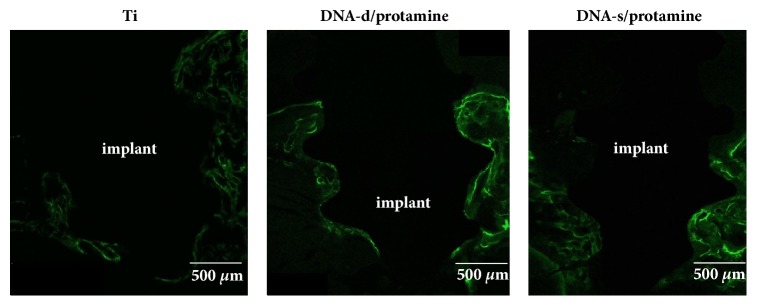
CLSM images of Ti, DNA-d/protamine, and DNA-s/protamine implants in the maxillary bone, performed with calcein 2 weeks after surgery.

**Figure 9:**
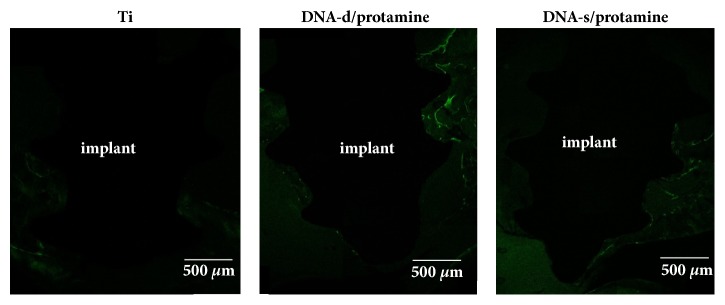
CLSM images of Ti, DNA-d/protamine, and DNA-s/protamine implants in the maxillary bone, performed with calcein 5 weeks after surgery.

The rats were maintained in good health during the test periods. Clinical signs of inflammation or adverse tissue reactions were not observed when the rats were killed. Typical CLSM images of Ti, DNA-d/protamine, and DNA-s/protamine implants after implantation (3 and 6 weeks) are shown in Figures 8 and 9, respectively. Green fluorochrome emission from calcein deposition was observed. At 3 weeks, DNA-d/protamine and DNA-s/protamine produced more distinct green labeling compared with that of Ti. The green labels of the three implants after 6 weeks were less visible and less distinct compared with those after 3 weeks. New bone formation was quantitatively evaluated according to the total length of calcein labeling in the ROI (Table 2). The calcein labeling lengths of DNA-d/protamine and DNA-s/protamine were significantly greater compared with those of the Ti implants 3 and 6 weeks after implantation (*p* < 0.05). There was no significant difference between DNA-d/protamine and DNA-s/protamine (*p* > 0.05). There were significant decreases in the lengths of the calcein labels in three different implants after 6 weeks compared with 3 weeks (*p* < 0.05).

### 3.3. Histological and Histomorphometrical Evaluations

**Figure 10:**
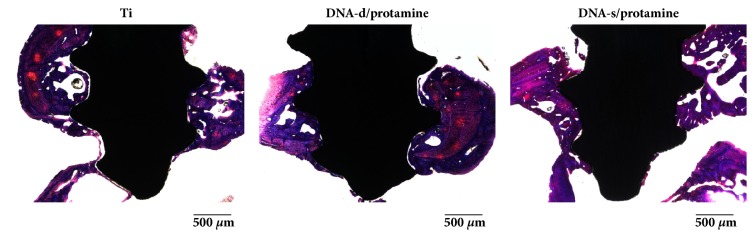
Histological appearances of Ti, DNA-d/protamine, and DNA-s/protamine implants 3 weeks after implantation.

**Figure 11:**
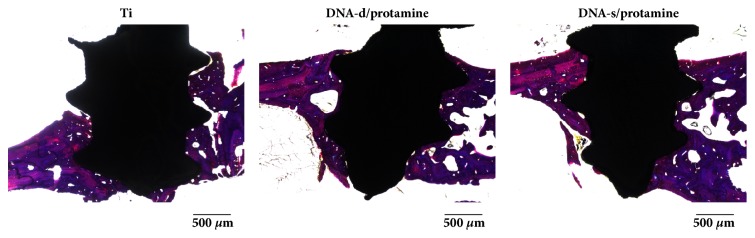
Histological appearances of Ti, DNA-d/protamine, and DNA-s/protamine implants 6 weeks after implantation.

The histological appearances of bone formation around the three different implants 3 weeks after implantation are shown in Figure 10. Failure or loosening of the implants did not occur during the preparation of histological samples, and severe inflammatory responses were not macroscopically observed in the tissues surrounding the implants. There was a distinct gap between the Ti implant and surrounding bone. Histological appearances 6 weeks after implantation are shown in Figure 11. Increased mature bone formation was observed at 6 weeks, and there were similar overall bone responses toward the three different implants.

The results of BIC measurements are shown in Table 3. BIC values of the DNA-d/protamine and DNA-s/protamine implants were significantly greater (*p* < 0.05) compared with those of the Ti implants 3 and 6 weeks after implantation. No significant differences were observed in BIC values between DNA-d/protamine and DNA-s/protamine 3 and 6 weeks after implantation (*p* > 0.05). The BIC values of Ti significantly increased from 3 to 6 weeks (*p* < 0.05), although there were no significant differences in BIC values of the DNA-d/protamine and DNA-s/protamine implants between 3 and 6 weeks (*p* > 0.05).

### 3.4. QCM

**Figure 12:**
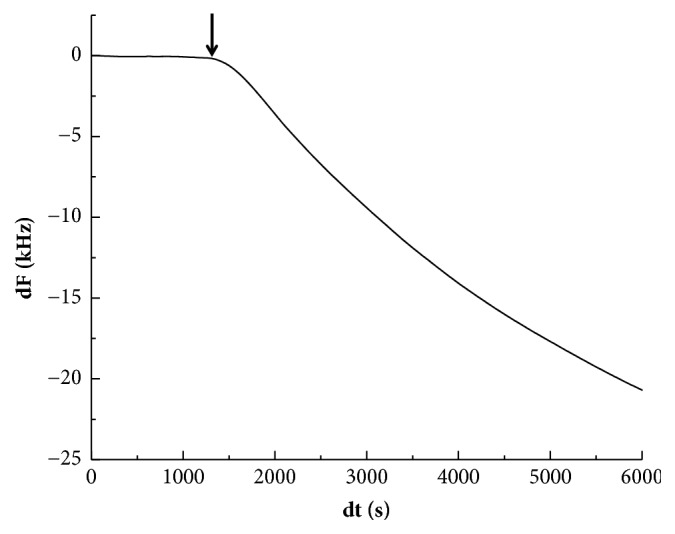
Typical frequency curves for the Ti sensor in SBF. The arrows indicate when the frequency began to decrease.

A typical frequency curve for Ti sensor is shown in Figure 12. A significant decrease was not initially observed, and the decrease in frequency was identified with time after the SBF injection (Figure 12, arrow). The decrease in frequency was detected after approximately 20 min for the Ti sensor and approximately 5–7 min for the DNA-d/protamine and DNA-s/protamine sensors.  Table 4 lists the time at which the frequency began to decrease and the estimated amounts of apatite formation after SBF injection. The time for DNA-d/protamine and DNA-s/protamine was significantly shorter than that of Ti (*p* < 0.05). Significant differences were not detected between DNA-d/protamine and DNA-s/protamine sensors (*p* > 0.05). There were no significant differences in the estimated amounts of apatite formation among the three sensors (*p* > 0.05).

### 3.5. WDS Analyses

**Figure 13:**
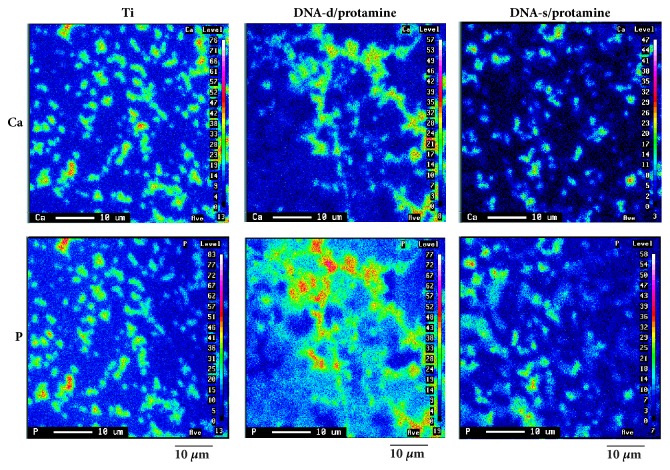
WDS mapping images of Ca and P on each sensor.

Figure 13 shows the elementary mapping distribution of Ca and P on Ti, DNA-d/protamine, and DNA-s/protamine sensor surface after the immersion in SBF. The presence of Ca and P was confirmed on each sensor.

## 4. Discussion

In the present study, we compared the bone response to titanium implants coated with DNA-d or DNA-s. DNA-d/protamine- or DNA-s/protamine-coated implants were inserted into the extracted sockets of rat maxillary molars. Moreover, apatite deposition in SBF was evaluated for each coated implant. We show here that DNA-d/protamine and DNA-s/protamine coatings enhanced new bone formation after the early stage of bone healing and that there was no significant difference between new bone formations for DNA-d/protamine or DNA-s/protamine. Both DNA coatings also promoted the initiation of apatite deposition in SBF immersion.

Measurement of the lengths of calcein labeling revealed that the DNA-d/protamine and DNA-s/protamine coatings stimulated new bone formation at 3 weeks. Sakurai et al. reported that the total length of calcein labels was almost the same 9 weeks after implantation of Ti and DNA-d/protamine coatings and suggested that bone healing was almost achieved at 9 weeks [21]. Raita et al. also reported the decrease of length of calcein labels from 3 weeks to 9 weeks for apatite and bisphosphonate coated Ti implants [26]. It is presumed that the decrease in the length of calcein labels at 6 weeks in the present study also suggested processing of normal bone remodeling in the tissue surrounding the implants.

Here we show that 3 and 6 weeks after implantation, the BIC values were significantly greater for both DNA coatings compared with that of the Ti coating. Moreover, the BIC values of both DNA coatings at 6 weeks were not significantly increased compared with those at 3 weeks, although there was a significant increase in the BIC values of the Ti implant after 6 weeks. Sakurai et al. reported that there was no significant increase in the BIC value of a DNA-coated implant after 9 weeks and speculated that bone healing was almost achieved at 9 weeks [21]. It is presumed that new bone formation was enhanced at early stage of bone healing process for both DNA coatings. On the contrary, in the case of Ti, new bone formation was suppressed at the early stage after the implantation and bone formation was progressed on Ti surface during 3 weeks to 6 weeks, although the mechanism of bone formation at the interface between bone and implant surfaces is not still clear. More detailed analysis at the interface between bone and implant surfaces should be needed.

The surface roughness and wettability of the materials influence bone responses [30, 31]. Generally, a rougher surface achieves a better bone response. Further, the hydrophilic surfaces of Ti promote early bone formation, osseointegration, or both in animal experiments [32, 33]. However, other surface characteristics such as topography, in concert with surface wettability, may induce a synergistic effect on biological responses. It is reported that surface treatments with increased roughness and hydrophobicity lead to an increased BIC value and higher removal torques during unscrewing, causing bone fractures compared with as-machined mini-implants [34]. Further, we show here that surface roughness and wettability influenced the improvement of bone formation of each DNA-multilayer coating.

The present QCM measurements simulate* in vivo* mineralization. Numerous studies show that the increased formation of apatite deposits after SBF immersion indicates better bone formation* in vivo *[35, 36]. Sakurai et al. also reported that DNA-d/protamine coating showed more amounts of apatite deposition on its surface than Ti only after 1 day immersion in SBF, but almost same amounts of apatite deposition to Ti after 7 days immersion [21]. It is presumed that apatite deposition occurred at earlier time after the immersion in SBF on DNA-d/protamine coating surface compared to Ti. Thus, we intended to make clear when apatite deposition will start. It is difficult to detect the time of beginning of apatite deposition by traditional immersion experiments. QCM method is a straight forward technique for monitoring the molecular behaviors such as adsorption or deposition on its surface by detecting the frequency decrease. When molecule bound on oscillating quartz crystal, oscillating frequency decreases are simply related to the binding amount of molecules on the crystal surface. QCM method can detect the adsorption or deposition of some molecules at nanoscale level in real time.

In the present study, we used 27-MHz QCM, which yielded highly sensitive measurements and decreased noise [37, 38]. HBSS was employed as SBF [28]. Yoshida et al. used a 27-MHz QCM technique to evaluate apatite formation on Ti and ZrO_2_ sensors in HBSS [24]. They found that apatite formation on the Ti surface occurred at earlier time compared with that on a ZrO_2_ surface, suggesting that earlier apatite formation may induce earlier bone formation on Ti compared with ZrO_2_. Present QCM results indicated that DNA-d and DNA-s coating surfaces produced earlier apatite deposition than Ti surface. However, estimated amounts of apatite formation were almost the same on Ti, DNA-d, and DNA-s coating surfaces. It is presumed that DNA coatings only influenced the initial nucleation stage for apatite deposition and not apatite growth stage. More detailed analysis for nucleation and growth stage for apatite deposition should be needed in the next QCM experiments.

Previous QCM measurements revealed that negatively charged surfaces with phosphoric or carboxylic acid terminal groups induce surface apatite formation in SBF [39, 40]. Apatite formation in SBF is initiated by calcium ion binding to a negatively charged surface. In the present study, the phosphate groups of DNA will bind calcium ions. Thus, it is presumed that the presence of phosphate groups in DNA-d and DNA-s contributed to early apatite formation in SBF and earlier apatite deposition on DNA-d and DNA-s coating surfaces related to early bone formation for both DNA coatings in animal experiments. However, protein attachment and cell phenotypes, such as adhesion, proliferation, and differentiation, influence* in vivo* bone formation besides apatite formation. The contribution of phosphate groups or the conformation of DNA required for protein attachment or cell phenotypes should be further investigated. Moreover, details about the steric conformation for coated DNA, for example the presence of steric hindrance, will be analyzed to elucidate the difference in bone response between DNA-d/protamine and DNA-s/protamine coating.

As mentioned above, DNA-s is used as nutritional supplement and is less expensive. It is expected therefore that the DNA-s/protamine coating will be more beneficial for clinical applications than the DNA-d/protamine coating. However, cytokines or antibiotics do not bind DNA-s. For example, loading BMP2 onto multilayered DNA coatings influences the behavior of osteoblast-like cells [41]. The effectiveness of the incorporation of cytokines or antibiotics into multilayered DNA coating will be studied in the future.

## 5. Conclusions

In the present study, we analyzed bone responses to titanium implants coated with DNA-d or DNA-s. After implantation into the extracted sockets of rat maxillary molars, we found that DNA-d/protamine- or DNA-s/protamine-coated implants promoted new bone formation at the early stage of bone healing. Apatite deposition in SBF measured using the QCM technique revealed early apatite formation onto DNA-d/protamine- and DNA-s/protamine-coated surfaces. We suggest that DNA multilayered coating is a useful technique to introduce a surface modification of a Ti implant for enhancing early bone formation.

## Figures and Tables

**Table 1:** Surface roughness (Sa) and contact angle (*θ*) of specimens.

Specimen	Sa (*μ*m)	*θ*(°)
Ti	0.30 (0.02)^a^	74.42 (3.91)^a^
DNA-d/protamine	0.64 (0.06)^b^	49.14 (5.80)^b^
DNA-s/protamine	0.53 (0.06)^c^	44.92 (9.35)^b^

Values in parenthesis indicate SD.

Different letters indicate significant difference in Sa or in contact angles among Ti, DNA-d/protamine, and DNA-s/protamine implants (*p *< 0.05).

**Table 2:** Total length of calcein labels in ROI (mm/mm^2^).

Implants	3 Weeks	6 Weeks
Ti	0.42 (0.16)^a,A^	0.25 (0.03)^c,B^
DNA-d/protamine	1.25 (0.40)^b,C^	0.60 (0.09)^d,D^
DNA-s/protamine	1.71 (0.25)^b,E^	0.56 (0.11)^d,F^

Values in parenthesis indicate SD.

Different small letters indicate significant differences among Ti, DNA-d/protamine, and DNA-s/protamine implants at 3 weeks or at 6 weeks (*p* < 0.05).

Different large letters indicate significant differences between 3 weeks and 6 weeks in the same implant material (*p* < 0.05).

**Table 3:** Percentage of the measured BIC (%).

Implants	3 Weeks	6 Weeks
Ti	20.5 (2.4)^a,A^	41.2 (14.2)^c,B^
DNA-d/protamine	55.3 (6.7)^b,C^	65.5 (7.4)^d,C^
DNA-s/protamine	59.8 (4.6)^b,D^	66.9 (4.4)^d,D^

Values in parenthesis indicate SD.

Different small letters indicate significant differences among Ti, DNA-d/protamine, and DNA-s/protamine implants at 3 weeks or at 6 weeks (*p* < 0.05).

Different large letters indicate significant differences between 3 weeks and 6 weeks in the same implant material (*p* < 0.05).

**Table 4:** Time at which frequency began to decrease and estimated amounts of apatite formation after SBF injection.

Specimen	Time (s)	Estimated amounts (*µ*g/cm^2^)
Ti	1139.0 (174.2)^a^	22.0 (5.3)^c^
DNA-d/protamine	424.0 (42.2)^b^	20.8 (8.8)^c^
DNA-s/protamine	314.7 (97.6)^b^	23.3 (4.6)^c^

Values in parenthesis indicate SD.

Different letters indicate significant difference in Times or Estimated amounts among Ti, DNA-d/protamine, and DNA-s/protamine implants (*p *< 0.05).
